# Noninvasive assessment of gastrointestinal parasites infection in free-ranging wild herbivores and adjoining livestock of Panna Tiger Reserve, Madhya Pradesh, India

**DOI:** 10.14202/vetworld.2017.748-751

**Published:** 2017-07-06

**Authors:** Abhay Sengar, A. B. Shrivastav, K. P. Singh, Amol Rokde

**Affiliations:** Centre for Wildlife Forensic and Health, Nanaji Deshmukh Veterinary Science University, Jabalpur - 482 001, Madhya Pradesh, India

**Keywords:** free ranging wild herbivores, gastrointestinal parasites, Panna Tiger Reserve

## Abstract

**Aim:**

This study was conducted to know the epidemiology of gastrointestinal parasites of free-ranging wild herbivores and adjoining livestock of Panna Tiger Reserve, Madhya Pradesh, India.

**Materials and Methods:**

A total of 374 fecal samples from wild herbivores (Chital *Axis axis* - 123, Sambar *Rusa unicolor* - 94, Nilgai *Boselaphus tragocamelus* - 86, and Chinkara *Gazella bennettii* - 71) and 284 fecal samples of domestic herbivores (cattle - 118, buffalo - 78, and goat - 88) were collected from common grazing land and adjoining area of tiger reserve. Detailed coprological examination for the presence of parasitic eggs/oocysts by direct smear examination, standard sedimentation, and floatation techniques was performed.

**Results:**

Fecal samples (n=374) of four different species of wild herbivores were screened. Out of which, 55.61% (n=208) were positive for parasitic infection. Among them, 13.10% (n=49) were positive for mixed parasitic infection of two or more parasite and 42.5% (n=159) were found positive for single parasitic infection. A total of 284 fecal samples of domestic animals were screened from adjoining areas of the tiger reserve. Out of which, 66.54% (n=189) were positive for parasitic infections, out of which 19.71% (n=56) were positive for mixed infection of two or more parasites, and 46.83% (n=133) were found positive for single parasitic infection.

**Conclusion:**

Wild herbivores at Panna Tiger Reserve were exposed to parasites including some that are known to be pathogenic; majority of wild animals had mixed infection of *Eimeria* spp., *Trichuris* spp., *Moniezia* spp., *Amphistome*, *Strongyloides* spp., *Balantidium* spp., and *Fasciola* spp.

## Introduction

Pathogen maintenance within wildlife populations and spill-over to livestock has been reported as a precursor to disease emergence in humans [1,2]. Diseases have been documented as a major cause of local extirpation of a number of wild animal species in India. Until and unless different epizootiological cycle of various parasitic infections is delineated and it is difficult to plan out measures to eradicate these diseases from free-ranging wild animals [3].

With the advancement of agriculture and cattle-raising into natural areas, humans and their domestic animals have recently been coming into greater contact with populations of wild animals in their habitats. This closer contact facilitates the spread of infectious agents and parasites to new hosts and environments, thereby establishing new relationships between hosts and parasites, and new ecological niches in the disease transmission chain [4].

Wild animal under free range condition is susceptible to almost all diseases as other animals, particularly helminth infection. Parasites can affect host survival and reproduction directly through pathological effect (blood loss, tissue damage, spontaneous abortion, congenital malformation, and death) and indirectly by reducing the host immunity and affecting the physical condition. Through these proximate mechanism, parasite can potentially regulate host-pathogen [5].

The emergence of infectious diseases with zoonotic potential has dominated research on wildlife pathogens over recent years. As a result, not only studies on the biodiversity and ecology of parasites been neglected but also efforts to control them have been impaired. The research focus has been directed toward humans and domestic animals. However, there is also a need to obtain greater understanding of how these emerging pathogens interact with sets of organisms living together in wild ecosystems [6].

Infectious diseases are the third most important driver of population decline of wildlife [7] after hunting and habitat degradation. Diseases that are shared between species also represent a potential burden to the whole ecosystem, affecting biodiversity, changing behavior or composition of animal populations, and even relegating species to the fringe of extinction [8,9]. In livestock, economic losses are caused by gastrointestinal parasites in a variety of ways: They cause losses through lowered fertility, reduced work capacity, involuntary culling, a reduction in food intake, lower weight gain, lower milk production, treatment cost, and mortality in heavily parasitized animals [10].

## Materials and Methods

### Ethical approval

The research work was ethically approved by Institutional Animal Ethics Committee.

### Target area

This work was conducted at Pannatiger reserve, located in Panna and Chhatarpur districts of Madhya Pradesh on domestic and free-ranging wild herbivores. The tiger reserve is situated at a point where there is continuity of the tropical and subtropical dry broadleaf forest belt. The native fauna includes semi-captive elephant, tiger, leopards, chital, sambhar, nilgai, four-horned antelope, blackbuck, chinkara, barking deer, rhesus macaque, common languor, giant flying squirrel, gharial, sloth bears, hyena, wild boar, jackal, hare, porcupine, mongoose, and pangolin. This work core and buffer area was divided into three zones.

### Collection of samples

A total of 374 fecal samples from wild herbivores (Chital - 123, Sambhar - 94, Nilgai - 86, and Chinkara - 71) and 284 fecal samples of domestic herbivores (cattle - 118, buffalo - 78, and goat - 88) were collected from common grazing land and adjoining area of tiger reserve. About 20 g of freshly voided fecal sample of each animal is collected in an individually labeled polythene bag, and these samples were properly sealed, labeled with date, time and place, brought to Centre for Wildlife Forensic and Health Laboratory for detailed coprological examinationfor the presence of parasitic eggs/oocysts by direct smear examination, standard sedimentation by Baermann test and floatation techniques by Shatter sugar method [11,12].

### Statistical analysis

The analysis of data was performed using Chi-square test.

## Results

### Prevalence of parasitic infection in wild herbivores

Fecal samples (n=374) of four different species of wild herbivores were collected from three different zones and subjected for examination. Out of which, 55.61% (n=208) were positive for parasitic infection. Among them, 13.10% (n=49) were positive for mixed parasitic infection of two or more parasite and 42.5% (n=159) were found positive for single parasitic infection, whereas remaining (n=166) samples were found negative for parasitic infection. Among different parasitic infections in wild herbivores, the highest prevalence was recorded for (n=374) *Strongyloides* spp. (20.58%) followed by *Eimeria* spp. (11.76%), *Trichuris* spp. (11.22%), *Moniezia* spp. (9.89%), *Amphistome* (9.09%), *Balantidium* spp. (3.20%), and *Fasciola* spp. (0.80%).

### Prevalence of parasitic infection in domestic herbivores

A total of 284 fecal samples of domestic animals were collected from adjoining areas of the tiger reserve. Out of which, 66.54% (n=189) were positive for parasitic infections, out of which 19.71% (n=56) were positive for mixed infection of two or more parasites, and 46.83% (n=133) were found positive for single parasitic infection, whereas remaining (n=95) samples were found negative of parasitic infection. The overall prevalence rate of *Strongyloides* spp. was maximum (17.25%) followed by *Amphistome* (14.08%), *Eimeria* spp. (13.73%), *Trichuris* spp. (7.39%), *Balantidium* spp. (4.57%), *Moniezia* spp. (4.23%), and *Fasciola* spp. (0.00%).

### Comparative prevalence of parasitic infection in wild and domestic herbivores

Status of gastrointestinal parasites in between wild herbivores (n=374) and domestic herbivores (n=284) revealed *Strongyloides* spp. (20.58%), *Eimeria* spp. (11.76%), *Trichuris* spp. (11.22%), *Moniezia* spp. (9.89%), *Amphistome* (9.09%), and *Balantidium* spp. (3.02%) in wild herbivores. While the corresponding figures encountered for *Strongyloides* spp. (17.25%), *Eimeria* spp. (13.73%), *Trichuris* spp. (7.39%), *Moniezia* spp. (4.22%), *Amphistome* (14.08%), and *Balantidium* spp. (4.57%) in domestic herbivores.

In wild herbivores, least prevalence was found for *Fasciola* spp. (0.08%) while infection of *Moniezia* spp. (4.22%) was recorded in domestic herbivores. Statistical analysis revealed significant (p<0.01) corelation between parasitic infection of wild and domestic animals (Table-1).

**Table-1:** Comparative prevalence of parasitic infection in wild and domestic herbivores.

Parasite	Prevalence in wild animals n=374 (%)	Prevalence in domestic animals n=284 (%)	χ^2^
*Strongylidae*	77 (20.58)	49 (17.25)	6.06 S
*Trichuris.* spp	42 (11.22)	21 (7.39)	
*Amphistome*	34 (9.09)	40 (14.08)	
*Fasciola.* spp	3 (0.80)	0 (0.00)	
*Moniezia.* spp	37 (9.89)	4 (1.40)	
*Eimeria.* spp	44 (11.76)	14 (4.92)	
*Balantidium.* spp	12 (3.20)	13 (4.57)	

S=Significant (p<0.01)

The results of this study revealed that the wild and domestic animals were infected with one or the other topographically fluctuating parasites. The overall prevalence of parasitic infection was (55.61%) in native wild herbivores, whereas in domestic herbivores, it was (66.54%).

## Discussion

Sharing of same pasture land and water holes by wild and domestic animals significantly increases the prevalence of parasitic infection. This can be further supported by finding of Mandal *et al*. [13] documented that grazing area and water holes shared by cattle and free ranging chital in MudumalaiWildlife Sanctuary significantly increased the prevalence of parasitic infection. Singh *et al*. [14] performed the study in different wild herbivores of Van-vihar National park and found the eggs of *Strongyloides* spp. (26.15%), *Eimeria* (6.20%), *Fasciola* spp. (2.64%), *Amphistomes* (1.98%), and *Trichuris* spp. (1.84%).The variation in topographical location of the protected area appears to influence the rate of prevalence. The aforementioned studies were conducted in hillocks and swampy meadows where snail population, which serves as intermediate host for flukes, is abundant in natural water sources facilitating higher concentration of metacercaria, the infective larval stage, on the pasture. Com-paratively less research has been directed toward understanding the origins of animal diseases, particularly at the wildlife-livestock interface, as well as the associated impacts on each sector [15,16]. *Strongyloides* were found to most prevalent parasites in this study which could be due to more conducive environment for the development of the preparasites stages in the hot and humid environmental condition of this region [17]. Moderate temperature and more humidity between the soil and the herbage favorable to the survival of eggs and free-living stages of parasites. The higher rate of prevalence during the rainy season is due to the existence of a suitable microclimate for the survival and propagation of free-living larval stages of parasites at several places. The parasites ova, snails, and other intermediate host get a favorable humid sub-tropic climate for development in the plane grazing areas with shallow temporary stagnated water. The animals congregate at the greens available around the periphery of such areas and naturally acquire more infection [16]. Endoparasite fauna in wild animals and consequent detection of infection in these animals might suggest that there could be proximity to and interactions with domestic animals [18]. The high prevalence encountered may be explained by the existence of favorable climate condition which supports prolonged survival of infective nematode larvae on pasture [19]. In this study, we also reported wild herbivores and livestock are not came to close contact, but they share same pasture for grazing.

## Conclusion

This study revealed that the prevalence of gastrointestinal tract (GIT) parasites infection in wild herbivores and adjoining livestock at Pannatiger reserve. The intensity of infestation by GIT nematodes also varies from no to heavy infestation. Management of diseases is an important component to wildlife conservation, considering that most species are already threatened due to habitat fragmentation and loss, diminished genetic diversity, overexploitation of herbivores themselves or their predators, and persecution by humans. Wild animals are also susceptible to lethal or debilitating pathogens, and coinfections can exacerbate clinical disease. Wild herbivores at Pannatiger reserve were exposed to parasites including some that are known to be pathogenic; however, most of the animals were in good physical condition. Parasitic prevalence is an important parameter to monitor the health of free-ranging wild herbivores. Future studies are required to evaluate the impact of GI parasites in the study area.

## Authors’ Contributions

AS and ABS designed the experiment. Analysis and the study was conducted by AS under the guidance of ABS. KPS and AR helped in collection of samples from field, execution of work and edit the manuscript. All authors read and approved the final manuscript.

## References

[1] Morse S.S, Mazet J.A.K, Woolhouse M, Parrish C.R, Carroll D, Karesh W.B, Zambrana-Torrelio C, Lipkin W.I, Daszak P (2012). Prediction and prevention of the next pandemic zoonosis. Lancet.

[2] Jones K.E, Patel N, Levy M (2008). Global trends in emerging infectious diseases. Global trends in emerging infectious diseases. Nature.

[3] Shrivastav A.B (2001). Wildlife health-a new discipline essential for tiger conservation programme. IntasPolivet.

[4] Correa S.R, Passos E.C, Fowler M.E, Cubas Z.S (2001). Wild animals and public health. Biology, Medicine, and Surgery of South American Wild Animals.

[5] Thawait V.K, Maiti S.K, Dixit A.A (2014). Prevalence of gastro-intestinal parasites in captive wild animals of Nandan Van Zoo, Raipur, Chhattisgarh. Vet. World.

[6] Thompson R.C.A, Lymbery A.J, Smith A (2010). Parasites, emerging disease and wildlife conservation. Int. J.Parasitol.

[7] Bengis R.G, Leighton F.A, Fischer J.R, Artois M, Morner T (2004). Therole of emerging and re-emerging zoonosesscientific and technical review world. Organ. Anim. Health.

[8] Williams E.S, Yuill T, Artois M, Fischer J, Haigh S.A (2002). Emerging infectious diseases in wildlife. Rev. Sci. Tech.

[9] Daszak P, Cunningham A.A, Hyatt A.D (2000). Emerging infectious diseases of wildlife-threats to biodiversity and human health. Science.

[10] Fikru R, Teshale S, Reta D, Yosef K (2006). Epidemiology of gastrointestinal parasites of ruminants in Western Oromia, Ethiopia. J. Appl. Res. Vet. Med.

[11] Soulsby E.J.L (1982). Helminths, Arthopods and Protozoa of Domesticated Animals.

[12] Sloss M.W, Kemp R.L, Zajac A.M (1994). Veterinary Clincal Parasitolgy.

[13] Mandal P, Jayathangaraj M.G, John L, Latha B.R, Raman M (2002). Prevalence of helminthic infection in free ranging chital (*Axis axis*) at Mudumalaiwildlife sanctuary, Tamil Nadu. XIII National Congress of Veterinary Parasitology, 14-16^th^ February, Kolkata.

[14] Singh S, Shrivastav A.B, Sharma R.K (2009). The epidemiology of gastrointestinal parasitism and body condition in free ranging herbivores. J. Threat. Taxa.

[15] Kock R (2014). Drivers of disease emergence and spread: Is wildlife to blame?On-derstepoort. J. Vet. Res.

[16] Voyles J, Kilpatrick A.M, Collins J.P, Fisher M.C, Frick W.F, McCallum H, Willis C.K, Blehert D.S, Murray K.A, Puschendorf R, Rosenblum E.B, Bolker B.M, Cheng T.L, Langwig K.E, Lindner D.L, Toothman M, Wilber M.Q, Briggs C.J (2015). Moving beyond too little, too late: Managing emerging in-factious diseases in wild populations requires international policy and partnerships. Ecohealth.

[17] Mir A.Q, Dua K, Singla L.D, Sharma S, Singh M.P (2016). Prevalence of parasitic infection in captive wild animalds in BirMotiBagh mini zoo (Deer park), Patiala Punjab. Vet. World.

[18] Holsback L, Cardoso M.J.L, Fagnani R, Patelli T.H.C (2013). Natural infection by endo-parasites among free-living wild animals. Rev. Bras. Parasitol. Vet.

[19] Allwin B, Balakrishnan S, Kumar N.V, Jayathangaraj M.G, Vedamanickam S, Gopal S (2016). Prevalence of gastrointestinal parasites in Gaur (*Bosgaurus*) and domestic cattle at interface zones of the Nilgiri Hills, Tamil Nadu, India. J. Vet.Sci. Technol.

